# One-Shot Synthesis of Thermoplastic Polyurethane Based on Bio-Polyol (Polytrimethylene Ether Glycol) and Characterization of Micro-Phase Separation

**DOI:** 10.3390/polym14204269

**Published:** 2022-10-12

**Authors:** Yang-Sook Jung, Sunhee Lee, Jaehyeung Park, Eun-Joo Shin

**Affiliations:** 1 Department of Organic Materials and Polymer Engineering, Dong-A University, Busan 49315, Korea; 2 Department of Bio-Fibers and Materials Science, Kyungpook National University, Daegu 41566, Korea; 3 Department of Fashion Design, Dong-A University, Busan 49315, Korea

**Keywords:** thermoplastic polymer, bio-based polyurethane, polymerization, biomaterials, micro-phase separation

## Abstract

In this study, a series of bio-based thermoplastic polyurethane (TPU) was synthesized via the solvent-free one-shot method using 100% bio-based polyether polyol, prepared from fermented corn, and 1,4-butanediol (BDO) as a chain extender. The average molecular weight, degree of phase separation, thermal and mechanical properties of the TPU-based aromatic (4,4-methylene diphenyl diisocyanate: MDI), and aliphatic (bis(4-isocyanatocyclohexyl) methane: H_12_MDI) isocyanates were investigated by gel permeation chromatography, Fourier transform infrared spectroscopy, atomic force microscopy, X-ray Diffraction, differential scanning calorimetry, dynamic mechanical thermal analysis, and thermogravimetric analysis. Four types of micro-phase separation forms of a hard segment (HS) and soft segment (SS) were suggested according to the [NCO]/[OH] molar ratio and isocyanate type. The results showed (a) phase-mixed disassociated structure between HS and SS, (b) hydrogen-bonded structure of phase-separated between HS and SS forming one-sided hard domains, (c) hydrogen-bonded structure of phase-mixed between HS, and SS and (d) hydrogen-bonded structure of phase-separated between HS and SS forming dispersed hard domains. These phase micro-structure models could be matched with each bio-based TPU sample. Accordingly, H-BDO-2.0, M-BDO-2.0, H-BDO-2.5, and M-BDO-3.0 could be related to the (a)—form, (b)—form, (c)—form, and (d)—form, respectively.

## 1. Introduction

Nowadays, thermoplastic polyurethanes (TPUs) are one of the most consumed families of polymers worldwide. These unique polymeric materials with a wide range of physical and chemical properties are broadly used in paints, coatings, synthetic rubbers, foams, fibers, adhesives, and packaging and in numerous fields such as the automotive industry, consumer or domestic equipment, construction engineering and biomedical applications [1,2,3]. The performances and properties of TPUs depend on the chemical nature of the reacting components and the utilized processes. Thus, by tailoring these factors, the TPUs can exhibit many useful properties, including modulable flexibility, elasticity, strength, good abrasion resistance, and high transparency [4]. TPUs are characterized by a segmented-block structure that is composed of a hard segment (HS; adduct of isocyanates and chain extender) and a soft segment (SS). Aliphatic and aromatic isocyanates are used to synthesize TPUs [5]. In addition, the choice of chain extender determines the characteristics of the HS and predominantly the physical properties of the TPU, and a low-molecular-weight diol or diamine is mainly used. The most important chain extender for the TPU is linear glycols such as 1,3-propandiol (PDO) and 1,4-butanediol (BDO). A SS introduces flexibility consisting of polyols (polyether or polyester polyols). Polyols constitute more than half of the total composition of the TPU and poly(ethylene glycol), poly(propylene glycol), poly(tetramethylene glycol) and poly(tetrahydrofuran) are commonly used polyether polyols, which are petrochemical-based. The synthesis of TPUs, including isocyanates, polyols, catalysts, and additives, and their application require abundant volatile organic compounds and emit hazardous pollutants that cause severe environmental pollution [6,7]. Since the application of TPU is increasingly extensive, decreasing the petrochemical monomers and energy required for polymerization and processing is becoming significant. The chemical industry is exploring new solutions based on natural raw materials, and, recently, the growing interest in applying bio-based substrates as a primary component in TPU synthesis is rapidly increasing. The new trend involves the replacement of petrochemical polyols and chain extenders used in synthesizing TPUs with materials obtained from biomass, including polysaccharides [8], sugars (such as xylose, mannose, glucose, galactose, and idose) [9,10], lignin [11], and vegetable oils [12,13] (such as soybean oil [14], castor oil [15], rubber seed oil [16], and palm oil [17]. Although vegetable oils are triglycerides, the esterification product of glycerol with three fatty acids, they do not possess suitable hydroxyl groups for polyurethane manufacture in many cases [4]. Moreover, after chemical modification, most vegetable oil-based polyols contain more than two hydroxyl groups or free long-chain fatty acids, which limits their application as the starting material for TPU elastomers [18]. Currently, bio-based polyols are used often for polymer production. This study concerns bio-based TPUs obtained using 100% bio-based polyether polyol, PO3G (Poly trimethylene ether glycol, SK Chemical). This polymer is linear with a molecular weight of 1000 g/mol and has excellent biodegradability and thermal and oxidation stability [19].

Using a diisocyanate, polyol, and low-molecular chain extender, the TPUs can be synthesized using two methods, particularly a one-shot and a pre-polymer process [20]. In the pre-polymer process, polyols and isocyanates first react to form an isocyanate-terminated pre-polymer, followed by the second step of chain extension by adding the chain extender. Numerous TPU elastomers and all polyurethane-ureas are prepared via NCO pre-polymer intermediates. This method allows the complete reaction (even of low-reactivity polyether diols) in the absence of catalysts and the intentional preparation of a segmented structure. Conversely, in the one-shot process, the starting materials are mixed in a reactor, and polymerization is performed. This process, which is conducted without solvents, is generally rapid, particularly in the presence of catalysts. Therefore, formed materials, for example, are prepared to utilize the one-shot process by mixing the reactants directly with additives [21]. The reaction is exothermic and is substantially terminated within 2–30 min, depending on the catalyst applied.

TPUs are block copolymers with a specific organization of HSs and SSs, and this segmented structure is the key feature of the TPU molecular chain [22,23]. The HS (may be glassy or semicrystalline) is typically composed of a rigid diisocyanate and a chain extender (for example, a short polyol) [24,25]. However, even for isocyanate compounds with the same NCO, each compound has a different molecular shape and property. Hence, the fields wherein each compound is applied are also different. Due to the thermodynamic incompatibility between the two structural units, the polymers undergo microphase separation resulting in HS domains dispersed in the SS matrix [26,27]. Moreover, the incompatibility results in a characteristic microphase-separated structure of TPU, with hard domains acting as the tie points for the flexible SS phase. The microphase separation of these two chemically distinct components generates unusual and useful physical and mechanical properties of TPUs. This phase separation between HSs and SSs occurs rapidly and simultaneously with polymerization and results from the thermodynamic immiscibility between the rigid and soft phases, forming microphases bound together by hydrogen bonding [28,29]. Since a wide range of monomeric materials is now commercially available, extensive investigations have been devoted to the structure-property relationships of TPUs, and tailor-made properties can be obtained from well-designed combinations of monomeric materials. The lengths and chemical structures of HS and SS, including the soft/hard segment [30,31], diisocyanate symmetry [25,32], ability hydrogen bonding ability, and crystallinity of soft/hard phase domains, and the synthesis and processing methods [33,34] are important features that determine the phase separation and physical properties of TPUs. In TPUs, the observed domain morphology and microphase separation are strongly related to the hydrogen bonding of HSs and their crystallization kinetics [3,35]. The structures and properties of TPUs are known to change as a function of temperature. These changes have been extensively investigated by thermal analysis [36,37], spectroscopy [38,39], dynamic mechanical analysis [21,25,32], wide-angle X-ray diffraction [30,40], and small-angle X-ray scattering [41]. Numerous studies have reported the relationship between the mechanical and thermal properties and the microphase structure of TPUs [42,43]. Although the combination of HS and SS of the TPU can have various morphologies depending on the synthesis conditions, studies that clearly define the difference in the morphology according to the synthesis conditions are insufficient. Therefore, in this study, we presented various models according to the [NCO]/[OH] molar ratio and isocyanate type. The present contribution is aimed at TPU synthesis based on bio-based polyol using the one-shot process and controlled microphase separation structure according to the HS type (diisocyanate symmetry) and HS contents. Thus, this study focuses on: (1) the effect of increased bio-content on the synthesis and (2) the effect of SSs or HSs (with different [NCO]/[OH] molar ratios, isocyanate type (MDI, H_12_MDI)) on the surface (morphological behavior and hydrogen bonding), crystallinity, molecular weight, and thermodynamical and mechanical properties. Through this analysis, (3) several types of micro-phase-separated in a two-phase system as the HS and SS matrix were modeled, depending on the [NCO]/[OH] molar ratio and the isocyanate type when synthesizing TPU. The combination of spectroscopy, X-ray scattering, microscopy, and thermal and mechanical analyses was used to characterize these complex materials in detail.

## 2. Materials and Methods

### 2.1. Synthesis of Bio-Based Thermoplastic Polyurethanes

**Figure 1:**
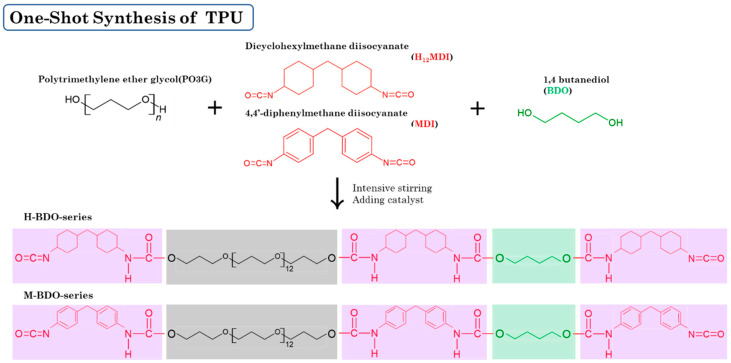
A schematic representation of the bio-based thermoplastic polyurethane (TPU) synthesis.

We prepared different segmented TPUs by changing the [NCO]/[OH] molar ratio and content of the HS. The HS consisted of 4,4-methylene diphenyl diisocyanate (MDI) and dicyclohexylmethane diisocyanate (H_12_MDI) as diisocyanate and BDO as the chain extender; the SS consisted of PO3G. TPUs were synthesized using a solvent-free one-shot polymerization procedure (Figure 1). The molar ratio of the OH groups of the polyol, NCO groups of the MDI or H_12_MDI, and OH groups BDO was maintained at 1:2.0:1, 1:2.5:1, and 1:3.0:1, respectively, and the resulting TPUs were identified (Table 1). The MDI that had been frozen was melted in the oven at 80 °C for 4 h. In the one-shot method, the PO3G polyols (M_n_ = 1000) and the chain extender BDO were thoroughly mixed in polypropylene beakers using a mechanical stirrer, which was then placed in the oven to maintain the temperature of the reaction mixture at 80 °C. Next, diisocyanate H_12_MDI or MDI and the catalyst dibutylin dilaurate (0.03 wt%) were added to the reaction mixture and mechanically stirred at room temperature (20–25 °C) for 1–2 min. As the mixture was stirred and polymerized, the transparent liquid became opaque, and the TPU was subsequently obtained. The reaction mixture was then poured into a Teflon-coated pan, cured in an oven at 100 °C for 24 h, and then kept to complete polymerization.

The final product was pressed at 180 °C for 5 min to obtain a film. All bio-based TPU films with 0.5–1 mm thickness were shown transparent white, and the light transmittance values of the H_BDO- and M_BDO-series ranged from 60–73% and 35–68%, respectively. After cooling to room temperature, the sheet was removed from the mold and used for further structural, thermal, and mechanical testing and characterization. All the reagents were of analytical grade and used without further purification. Detailed information on the materials is listed in Table 2.

### 2.2. Characterization

#### 2.2.1. Molecular Characteristics

The number average (M_n_) and weight average (M_w_) molecular weights and the polydispersity index (PDI) were measured by gel permeation chromatography (GPC) using a Viscotek GPCmax (VE-2001 system, Malvern, Worcestershire, UK). To test the solubility of the synthesized bio-based TPU in organic solvents, THF was used and applied to a mobile phase solvent. The column was 300 × 810 mm and the flow rate was 1 mL min^−1^ at 60 °C.

#### 2.2.2. Fourier Transform Infrared Spectroscopy (FT-IR) 

Fourier transform infrared (FT-IR) analyses were performed using a Nicolet Nexus FT-IR spectrometer (PerkinElmer, Shelton, CT, USA) over the wavelength range of 400–4000 cm^−1^, equipped with an attenuated total reflectance accessory.

#### 2.2.3. Atomic Force Microscopy (AFM)

The surface morphology of TPU films was investigated using an Inova system (Bruker, Billerica, MA, USA), equipped with a standard silicon nitride probe, SuperSharpSilicon™-SPM-Probe (NanoSensors™, Zurich, Switzerland; spring constant 42 N and resonant frequency 320 kHz). The analyses were performed under ambient conditions using the tapping mode atomic force microscopy (AFM) technique, and the surface images were taken in the sizes of 20 × 20 μm. The bulk morphology was evaluated by imaging the fracture area after the previous freeze-fracturing of sheets at a temperature of −80 °C. The AFM images were processed using NanoScope analysis software.

#### 2.2.4. X-ray Diffraction (XRD)

X-ray diffractograms were collected using a Shimadzu diffractometer (XRD-6000) with mono-chromatic CuKα radiation (λ = 0.15418 nm) and a generator working at 40 kV and 30 mA. Intensities were measured in the range of 5 < 2θ < 40°, typically with scan steps of 0.05° and 2 s/step (1.5° min^−1^). Peak separations were performed by Gaussian deconvolution.

#### 2.2.5. Dynamic Mechanical Analysis (DMA) 

Dynamic mechanical properties of the TPU films were determined using a DMA Q800 analyzer (TA instruments, New Castle, DE, USA) in the tensile mode at a frequency of 1 Hz. The samples were initially cooled to −100 °C and subsequently heated to 150 °C at a heating rate of 4 °C/min.

#### 2.2.6. Differential Scanning Calorimetry (DSC) 

The thermal properties of the obtained samples were measured on a DSC 8500 thermal analyzer (TA Instrument, New Castle, DE, USA). All the samples were weighed between 2 and 10 mg. The measurement was conducted from −70 °C to 250 °C at a heating rate of 20 °C/min under a nitrogen purge.

#### 2.2.7. Thermogravimetric Analysis (TGA) 

The thermal properties of the obtained samples were also determined using TGA Q500 (TA Instrument, New Castle, DE, USA), which were measured at a temperature range of 40–650 °C with a ramp heating rate of 10 °C/min in the presence of a nitrogen atmosphere. The weight of each sample was approximately 5 mg. The weight loss of 5% and 50%, the maximum degradation rate, and ash residue at 600 °C were registered.

#### 2.2.8. Shore A Hardness

Hardness was measured at room temperature using a Zwick Roell GS-706N analogical hard-ness testing apparatus (Teclock Co., Tokyo, Japan) using the “UNE-EN ISO 868:1998: Plastics and ebonite—Determination of indentation hardness by means of a durometer (Shore hardness)” standard procedure at (23 ± 2) °C and 50% relative humidity.

#### 2.2.9. Mechanical Properties 

Bio-based TPUs films were tested on an Instron 4201 autograph tester (Shimadzu, Tokyo, Japan) to measure the stress-strain behavior of the samples in tension. The length × width × thickness of the specimens was 10 × 2 × 0.5 mm.

## 3. Results and Discussion

### 3.1. Molecular Weight of Synthesized Thermoplastic Polyurethanes

The molecular weight of the synthesized TPUs was characterized by GPC analysis, and detailed measurements are summarized in Table 3. The M_n_ and M_w_ of the TPUs were in the range of 37,723–112,117 and 70,953–236,689, respectively. The M_w_ of TPUs strongly increased as the [NCO]/[OH] molar ratio increased. In addition, the number of urethane units in the hard domains increased with the [NCO]/[OH] molar ratio, resulting in higher molecular weights [3,44,45]. The M-BDO-series exhibited a higher molecular weight than the H-BDO-series, although it had a similar HS mole ratio, as shown in Table 3. Furthermore, the melting temperature increased as the M_w_ of TPUs was increased (the [NCO]/[OH] molar ratio was increased) (see Section 3.5). The HS melting temperature was found to depend on the HS–SS interaction and the number of hydrogen bonds in the HS [30,46]. Moreover, the M-BDO-series exhibited a high melting temperature because of its high M_w_. The M_n_ of the resulting bio-based TPUs was more than 30,000 g/mol, which is sufficiently high to satisfy the industrial application requirements and be the strong hydrogen-bonding character of TPUs.

The PDI values of the TPUs were in the range of 1.78–2.51, which is consistent with the molecular weights. The low PDI values indicated a narrow molecular weight distribution of the prepared TPU samples. However, the PDI values were approximately 2 (the theoretical value for linear step-growth polymers is 2 according to the Flory’s theory [47]) suggesting that the conversions of polymerizations were sufficiently high for the one-shot bulk reaction. In the case of M-BDO-2.0, the PDI was 1.95; thus, this condition is better for the one-shot bulk polymerization reaction with a short polymerization time because of the good control of the polymerization process. This is true for most TPUs, and it is nevertheless sufficient for polymer processing (injection and extrusion) and various applications [48]. These increasing values of M_n_ and M_w_ with increasing HS and using aromatic diisocyanate lead to differences in the TPU detected by FT-IR spectra, X-ray diffraction (XRD), AFM, DSC thermograms, and tensile strength and hardness, which are discussed below.

### 3.2. Chemical Structure Characterization (FT-IR)

**Figure 2:**
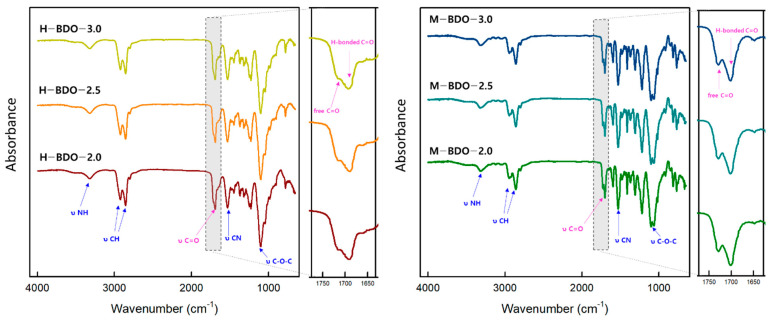
Fourier transform infrared (FT-IR) spectra of TPU-based H-BDO and M-BDO.

The chemical structure of the synthesized TPUs was confirmed through FT-IR spectroscopy. In Figure 2, the excess isocyanate can be detected using IR spectroscopy. The NCO band at 2270 cm^−1^ is one of the most intense bands and is practically undisturbed through the absorption of other groups. Therefore, the reaction is confirmed to be completed owing to the absence of absorption bands related to the NCO groups and at 3470 cm^−1^ corresponding to the OH groups of the polyol end group for all samples [49]. The N-H bond stretching vibration of urethane groups appeared at 3330 cm^−1^ due to hydrogen bonding. It is commonly known that the N-H bond can be observed in two separate bands, that is, the hydrogen-bonded N-H at 3275–3300 cm^−1^ and the free N-H bond at 3500 cm^−1^ [50]. The C-H asymmetric and symmetric stretching vibrations of the -CH_2_ groups were observed as bimodal bands with the maxima at 2850 and 2950 cm^−1^, respectively, which are assigned to the SS of the TPU matrix [32]. Bands 2900 cm^−1^ are assigned to the CH groups, particularly at 2950 cm^−1^ corresponding to the asymmetric CH_2_ stretching and the 2850 cm^−1^ band that is associated with the symmetric CH_2_ stretching [51]. Thus, TPU formulations with more content of SS exhibited bands with more intensity in this zone. The double peak observed in the 1680–1740 cm^−1^ range corresponds to the carbonyl group (C=O) stretching vibrations [52,53]. Further characteristic bands at 1600 cm^−1^ and 1819 cm^−1^ correspond to the C=C aromatic stretching vibration of the M-BDO-series. The band observed at 1530 cm^−1^ is associated with the stretching vibration of the –CN bond of the urethane groups. [35] The strong absorption band at 1104 cm^−1^ is ascribed to the free ether bond (C-O-C) of the used polyether polyol. The band maximum associated with the asymmetric stretching vibrations of the non-associated ether group is marked by the 1104 cm^−1^ band, while the 1063 cm^−1^ band is related to hydrogen bond interaction between N-H and C-O-C groups [33]. 

**Figure 3:**
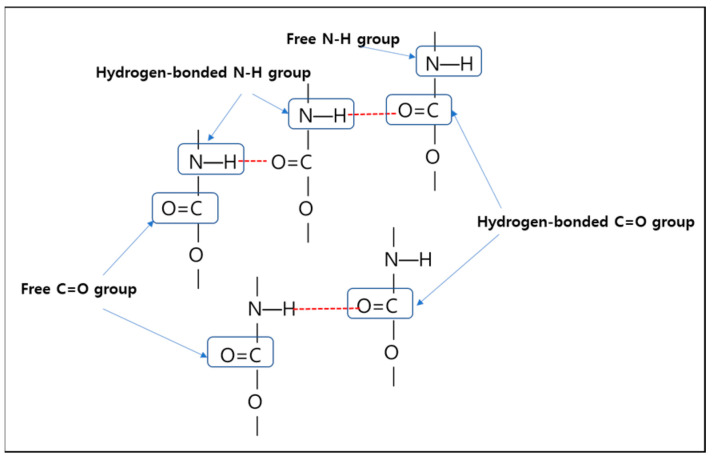
Specific hydrogen bonding in the TPUs.

FT-IR analysis is a useful method for characterizing the band intensity and shape of the localized vibrations associated with specific functional groups, N-H or C=O, which are involved in specific hydrogen bonding in various domains, as shown in Figure 3. The position and intensity of these vibrations are known to be susceptible to the strength and specificity of the formed hydrogen bond [22]. Thus, the phase separation in TPUs can be characterized by measuring the intensity and position of the hydrogen-bonded N-H stretching vibration. It is usually interpreted that extensive phase separation has occurred when there is significant N-H---O=C hydrogen bonding since both units are associated with the HS. It has also been suggested that N-H can form a strong hydrogen bond with the oxygen of the ether groups from polyol associated with the SS when available. The N-H bond vibration appeared in all synthesized TPUs regardless of the [NCO]/[OH] molar ratio. This region also exhibited a small shoulder at 3500 cm^−1^ in all the curves, corresponding to the non-hydrogen-bonded N-H group [23]. Furthermore, the band intensities in the hydrogen-bonding association of the carbonyl group (C=O) region can be utilized to characterize the phase separation between the HS and SS. The specific method to calculate the degree of phase separation (DPS) [24] is to analyze the spectra in the 1750–1680 cm^−1^ region by deconvolution of the carbonyl bands using Origin software (Origin 2018) in Table 4. In particular, the H-bonded C=O only existed in the HS, whereas free C=O was merely scattered in the SS. Therefore, the DPS was calculated based on the amounts of free carbonyl and hydrogen-bonded –C=O in the amorphous and ordered regions.

Degree of phase separation (DPS) = R/(R + 1) (1)

(2) $$R=\frac{\mathrm{Ab}(\mathrm{Absorption}\;\mathrm{intensity}\;\mathrm{of}\;\mathrm{hydrogen}\;\mathrm{bonded}\;C=O)}{\mathrm{Af}(\mathrm{Absorption}\;\mathrm{intensity}\;\mathrm{for}\;\mathrm{free}\;C=O)\;}$$

Degree of phase mixing (DPM) = 1 − DPS(3)

The exact band positions indicated only some differences among the TPUs containing different diisocyanates and [NCO]/[OH] molar ratios. These results indicate that, in the case of H-BDO and M-BDO, the DPS slightly increased with the [NCO]/[OH] molar ratio in correlation with the increasing HS content. The H-BDO-2.0 (R = 1.67) sample contained 55.5% of HS, which were connected with hydrogen bonds. For the H-BDO-3.0 sample (R = 1.40), slightly more hydrogen bonds were generated, with some limitations of the phase separation function depending on the diisocyanate type. Furthermore, increasing the amount of isocyanate to create the HS phase favored the elevation of the hydrogen bonding, as indicated by the corresponding increase in the R index. Compared to the H-BDO-3.0 sample, the M-BDO-3.0 sample exhibited high DPS despite having lower HS content. In the M-BDO-2.0 sample, it could be concluded that more than 57% of the HS was microphase separated, while only 42% of the HS was mixed within the polyether polyol matrix. Therefore, the amount of hydrogen-bonded C=O groups was affected by the HS content and diisocyanate type. In conclusion, DPS slightly increased for TPUs prepared with the MDI as diisocyanate. Factors influencing DPS in TPU materials include hydrogen bonding between polymer chains, segment length, polarity and crystallizability, overall composition, and mechanical and thermal history [25]. In the following section, the reason for phase separation between the HS and SS is discussed, and the experimental evidence supporting the presence of microdomains is presented.

### 3.3. Atomic Force Microscopy (AFM) Analysis

**Figure 4:**
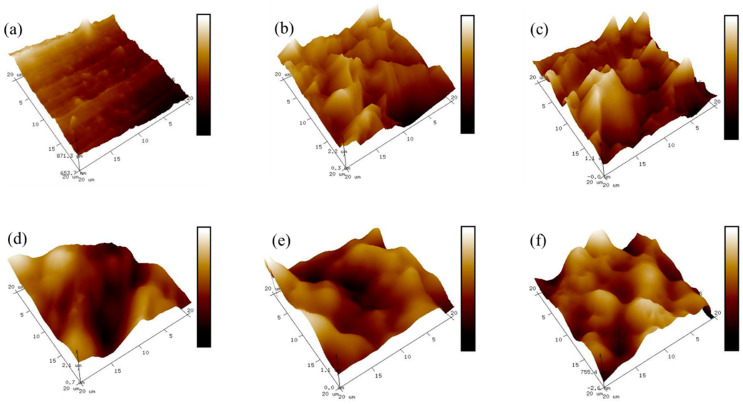
Atomic force microscopy phase images of the freeze-fractured surfaces of the bio-based TPU: (**a**) H−BDO−2.0, (**b**) H−BDO−2.5 (**c**) H−BDO−3.0, (**d**) M−BDO−2.0, (**e**) M−BDO−2.5, and (**f**) M−BDO−3.0.

The size and shape of the hard domains in the TPU were evaluated using AFM. Although AFM is typically used to analyze the surface physical structure and quantify the surface roughness [54,55,56], it is also a useful tool for the investigation of the internal structure of heterogeneous materials [32,57], particularly when other methods are inefficient due to low contrast or when a comparison with other analytical methods is desirable. The freeze-fractured cross-sections of the prepared bio-based TPU samples were analyzed to investigate whether the HS/SS morphology of the surface patterns of the films was irregular and relatively rough. Figure 4a–f shows the height and 3D AFM images of the TPU sample using the tapping mode, and Table 5 illustrates the supporting data of the AFM images. The samples exhibited two types of phase contrast: a dark, featureless matrix corresponding to the SS and bright elements of different sizes dispersed in this matrix [57]. The roughness increased when the HS content increased with the [NCO]/[OH] molar ratio, and the phase separation and hard domain structure became more pronounced in the sample morphology. The shape of the hilly was significantly different when the H-BDO- and M-BDO-series were compared. The H-BDO-series showed a sharp hilly, whereas a more rounded hilly was observed in the M-BDO-series. These characteristics are affected by the HS content and isocyanate type.

The samples containing a high SS content (H-BDO-2.0 in Figure 4a) exhibited smooth surfaces that were separated from each other in the samples. Conversely, the TPU with high hard domain content showed an irregular ‘‘hilly’’ break surface (Figure 4b–f). Dramatic changes were observed in the phase images when comparing H-BDO-2.5 to M-BDO-3.0 in Figure 4b,f, although the HS content was similar in both samples (41% of HS content). The M-BDO-3.0 sample showed the largest hilly surface with large globules and the highest roughness value. The depth and height of the globules in the z direction on the TPU surface were examined using Nanotec Electronica WSM software (2019). The roughness in the Z-profile of the TPU contributes to the co-continuous network morphology [58]. Various microdomains observed on the TPU surface could have significantly contributed to the roughness enhancement. The non-uniform distribution of the hard domain increased the surface roughness of the H-BDO-series from 23 to 228 nm. In addition, the surface roughness in the M-BDO-series was observed in a highly spiked region (R_max_ = 1872–4243 nm) from 178 to 347 nm. Consequently, variation in the size and shape of the hard domains depending on the isocyanate type can be confirmed. When the HS content increases by varying the [NCO]/[OH] molar ratio, the HS domains become larger (up to multi-µm size); the roughness and the extent of phase separation increase, and the hard domain structures become more pronounced and visible in the sample morphology. Many factors such as the [NCO]/[OH] molar ratio, HS content, and isocyanate type exhibiting diverse morphological structures are related to the AFM topographic images. Therefore, these factors support the DPS between the HS and SS and hydrogen bonding obtained by FT-IR analysis. When the HS content increases by varying the [NCO]/[OH] molar ratio, the HS domains become larger (up to multi-µm size); the roughness and the extent of phase separation increase, and the hard domain structures become more pronounced and visible in the sample morphology.

### 3.4. X-ray Diffraction (XRD) Analysis

**Figure 5:**
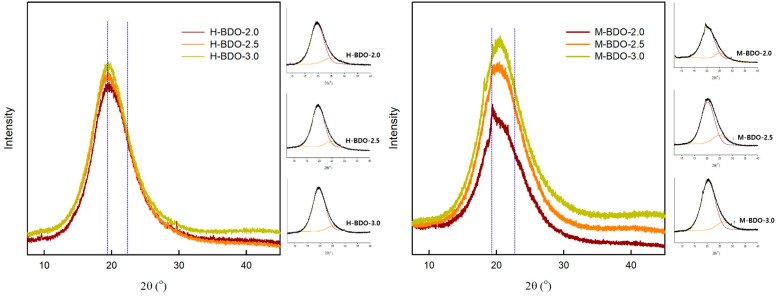
X-ray diffractograms of the bio-based TPU.

The degree of crystallinity in the prepared bio-based TPU was investigated by wide-angle XRD in the region of wider angles. The changes in the crystalline structure due to varying [NCO]/[OH] molar ratio and isocyanate type were determined by XRD analysis. A comparison of the XRD patterns with different HS contents is illustrated in Figure 5. The peaks in the TPU samples at 2θ = 19° generally correspond to the hard domain related to the hydrogen bonds between urethane groups in 2θ = 19–23° [59]. 

The observed diffraction patterns exhibited significant broadening of peaks, which resulted in two main hard domain regions with a maximum at approximately 19.4° and 23.5° by fitting with Gaussian distributions using Origin software (2018). The HSs demonstrated a diffraction peak at 19.4° with higher intensity when present in higher concentration. Furthermore, as the [NCO]/[OH] molar ratio was increased, the intensity of the peak localized at 2θ = 19.4° increased from 4858 to 5047 and 3355 to 5292 for the H-BDO- and M-BDO-series, respectively, with a sharper full width at half maximum (FWHM) in Table 6. This peak indicated a hard domain based on a higher-ordered arrangement of the HS with hydrogen bonding [41]. The phenomenon of the higher hard domain rate of the HS in M-BDO-3.0 attributed to the globular morphology of the hard phases, which was also confirmed by the AFM image in Figure 4f. 

In contrast, as the HS content increased, the diffraction peak at 23.5° exhibited a lower diffraction peak height and broader FWHM, indicating a smaller hard domain that decreased the peak intensity, which is also supported by the AFM images illustrating the decreased height of the surface in Figure 4a. Although a discrepancy was observed between the XRD and AFM results, AFM showed agglomeration, while a single aggregate containing a number of hard domains was observed in XRD. In segmented TPU, the phase separation of the SSs and HSs can occur depending on their relative contents, structural regularity, and thermodynamics incompatibility. The XRD studies revealed that the hard domain depends on the structure of diisocyanates and the [NCO]/[OH] molar ratio in the TPU hard domain. Moreover, Figure 4 shows that hard domain contents increased from aliphatic to aromatic characters of the diisocyanates utilized in the bio-based TPU.

### 3.5. Thermal Analysis (DSC)

**Figure 6:**
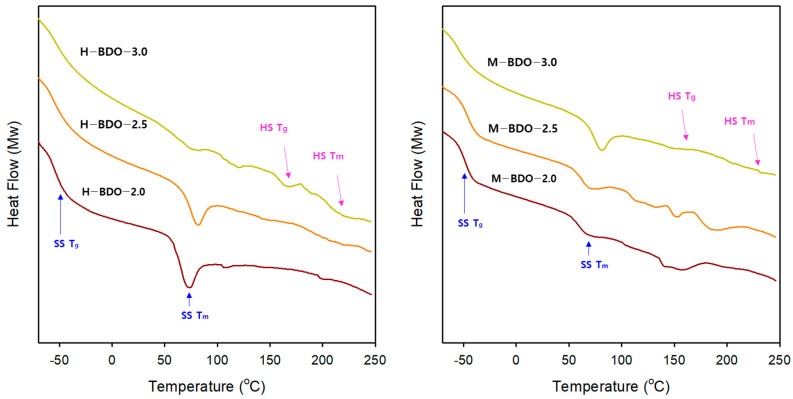
Differential scanning calorimetry (DSC) of the first heating ramp measured at 20 °C/min from −100 °C to 200 °C for the TPU-based sample series.

The thermal characteristics of the bio-based TPUs and their segmented structure were investigated using DSC. Regarding the thermal properties of TPUs, four types of thermal effects (the glass transition temperature of SS, melting temperature of SS, glass transition temperature of the amorphous part of the HS, and the melting of the HS) could be distinguished from the DSC runs (Figure 6), and the results are summarized in Table 7. The TPUs are segmented or block copolymers consisting of alternating HS and SS. The microphase separation of these two chemically distinct components gives thermodynamic incompatibility, generating separated peaks in T_g_ and T_m_ of the SS and T_g_ and T_m_ of the HS [60]. Generally, the HS and SS of a TPU are incompatible because they have a positive heat mixing. Thus, there is a tendency toward phase separation of the two components; however, the topology of the block copolymer molecules imposes restrictions on segregation, thereby forming a microdomain [61].

Up to four transitions were observed for the bio-based TPU in Figure 6, including the glass transition temperature (between −55 ℃ to −44 °C), the melting temperature of SS (67 °C to 80 °C), and the melting temperature of HS (160 °C to 231 °C). The SS T_g_ in the H-BDO- and M-BDO-series was detected at approximately −54 °C and −45 °C, respectively. H-BDO-2.0, H-BDO-2.5, and H-BDO-3.0 exhibited the melting of the SS at 72, 80, and 84 °C, respectively. The HS T_g_ in the M-BDO-series was detected at 136, 151, and 157 °C, while the melting temperature of the HS was observed at 160, 183, and 231 °C. When considering the [NCO]/[OH] molar ratio, the SS T_g_ of the H-BDO- and M-BDO-series increased with the [NCO]/[OH] molar ratio. The result of the H-BDO-series is explicated because of the increasing miscibility of the HS and SS, that is, the partial mixing of the HS within the SS matrix. The sample of the M-BDO-series demonstrated higher T_g_ of the SS as higher contents of the HS were well phase-separated, which increased the DPS as confirmed by the FT-IR analysis; this phenomenon may be an indicator of kinetically favorable and stable phase separation. Moreover, the T_m_ of SS slightly increased as the [NCO]/[OH] molar ratio was increased, while the heat capacity increased with increasing SS contents, which corresponded to the melting peak of the SS. Therefore, a TPU sample showing insignificant T_m_ of SS with higher melting enthalpy could be obtained. Since the T_m_ of SS is the deciding factor of the shape memory transition temperature, this result also suggested that the shape memory TPU can be adjusted to various temperature ranges by carefully selecting the [NCO]/[OH] molar ratio and isocyanate type, thus possibly expanding the application field. As an example of a shape memory polymer, H-BDO-2.0 exhibited a relatively sharp endothermic peak at approximately 72 °C, which resulted from the T_m_ of the SS-associated thermal transition temperature (T_trans_). When the temperature increase is higher than 72 °C (T_trans_) of the switching segments, the segments are flexible, and the polymer can be deformed elastically. The temporary shape is fixed by cooling below 72 °C, and the permanent shape is recovered when the polymer is heated again.

The length of the HS blocks forms the upper limit to the size of the HS crystals in the chain direction, which determines the melting point and thermal stability [62]. Figure 6 illustrates the temperature range wherein most HS melting temperatures shift towards higher temperatures as the HS content is increased. The T_m_ at higher temperatures corresponded to the dissociation of a great order structure, which was related to the mixing between the HS and SS. The lower temperatures could be attributed to the melting of the less-ordered structure or suitable SS [63,64]. In particular, the samples of the M-BDO-series exhibited increased high-temperature T_m_ peaks and their shift to higher temperatures, which resulted from better HS ordering and the formation of stronger and more stable HS domains. From a morphological viewpoint, the HS formed globular domains in a continuous SS matrix in this sample. The degree of order within the HS of the TPU depends on the chemistry, rigidity, and hydrogen-bonding within the HS [65]. In addition, the phase separation between the HS and SS depends on their respective lengths and affinity for each other, which is closely related to the ability of the HS and SS to establish hydrogen-bonding interactions. Therefore, the phase separation is affected by the chemical composition and HS content in the synthesized TPUs. The effect of the isocyanate type on the T_m_ of the TPUs was determined at a higher temperature using MDI. The materials obtained from aromatic isocyanates exhibited a higher glass transition temperature than those obtained from aliphatic isocyanates. The lower glass transition temperatures observed for TPUs obtained using aliphatic isocyanates are attributed to the better separations of the phases [34]. Conversely, the presence of aromatic isocyanate in the HS produces a stiffer polymer chain with a higher melting point.

### 3.6. Dynamic Mechanical Analysis (DMA)

**Figure 7:**
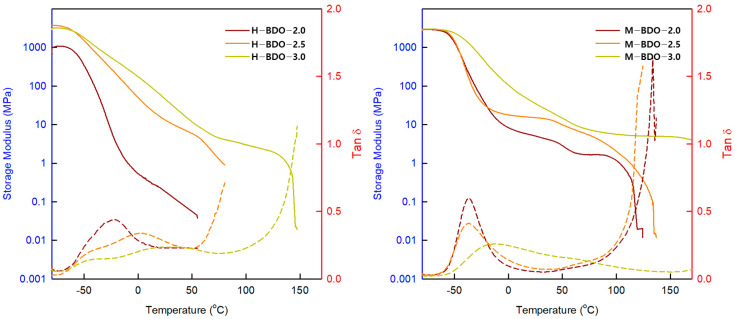
Storage modulus versus temperature and tan δ versus temperature for the obtained bio-based TPUs.

The DMA results are shown in Figure 7 as a functional relationship of the storage modulus (G’) and tan δ, revealing the thermal transitions and indicating the applicable temperature ranges. The storage modulus defines the energy stored elastically by the materials at deformation supplying information about the polymer stiffness [36]. The highest values of G′ were observed between 1085 and 2850 MPa for the H-BDO-series and 2918 and 3030 MPa for M-BDO-series. The G’ values below −50 °C remained nearly constant due to the restriction of the molecular motions to vibrations and short-range rotations of the SS. The gradual decrease in G′ at approximately −50 °C corresponded to the T_g_ of SS. The α-relaxation process, which is indicated by the low-temperature peak in the tan δ plots and represented the glass transition of the SS, was broad, suggesting only fair phase separation between SS and HS. In addition, this peak was sufficiently defined for the M-BDO-series, which allowed the assignment of the T_g_ based on the position of the peak maximum. The difference in the shape of the SS T_g_ peak is attributed to higher DPM in the H-BDO-series with high DPM, as indicated by the FT-IR analysis. In tan δ curves, the double peaks, which suggest the existence of the two phases of the SSs related to the SS T_g_ (approximately −54.77 °C to −40.56 °C) and SS T_m_ (approximately 1.35 °C to 48.23 °C), appeared in H-BDO-2.5 and H-BDO-3.0. The temperature difference between the above double peaks decreased as the [NCO]/[OH] ratio increased, particularly for the H-BDO-series. This result indicates that as the ratio of the HS increases, a sharp increase in tan δ appears due to the relaxation of the hard domain of the TPU molecules related to the presence of more HS in the soft domains that restrict the mobility of the SS. The T_g_ of TPU can be detected by DSC and DMA, and the obtained T_g_ values were similar. Since the HS content increased, the modulus, T_flow_ due to the melting of the HS, and the T_g_ increased.

The G’_25_ storage modulus significantly increased with the HS content in Table 8. In the case of phase-separated HS, the modulus considerably increased with the higher HS content, making the materials stiffer. However, no melting transition was detected in the DSC thermogram, whereas the flow behavior of the TPU could be observed in DMA. The modulus above the T_g_ of the SS in the rubbery plateau region depends on the reinforcing effect of the HS on the soft matrix [66]. The flex temperature (T_flex_) is defined as the temperature at the beginning of the rubber plateau region, that is, the intercept of the tangents. The flow temperature (T_flow_) is defined as the temperature where the storage modulus G’ reaches 1 MPa, and the storage modulus of the rubbery plateau is determined at room temperature. The modulus of the rubbery plateau, in the T_flex_ − T_flow_ region is a function of the HS crosslink density and reinforcement by the separated HS. Usually, TPUs from segmented copolymers can be prepared with a range of rubbery moduli by changing the HS content [32]. With the [NCO]/[OH] molar ratio, the T_flow_ of the corresponding TPU is increased from 77.7 to 170 °C. These T_flow_ values are attributed to the melting of the phase-separated HS, and the increase in T_flow_ observed may result from an increase in the hydrogen bonding in the HS domain [30]. The T_flow_ decreased with the decreasing HS content, which is also in agreement with the results observed in other systems. The T_flow_ of the MDI-based TPU was considerably higher than that of the H_12_MDI, which is also explained in the solvent effect theory by Flory [47]. In the MDI-based TPU, the rubbery plateau was extended and exhibited a higher rubbery elastic modulus and sharper peak compared to those of the H_12_MDI-based TPU. The constant value of this storage modulus indicates that no phase transitions occurred within this temperature range, and phase separation is effective. This result is attributed to higher DPS, which exists in the samples of the M-BDO-series, as indicated by the FT-IR results.

The MDI-based TPU demonstrated high values for T_g_, G’_25_, T_flex_, and T_flow_ compared to those of H_12_MDI-based TPU, suggesting a higher degree of stiffness of this sample. In addition, the regions of the rubbery plateau between T_flex_ and T_flow_ were extended. Typically, phase separation occurs in TPU materials because of the thermodynamic incompatibility between the SS and HS, resulting in elastomeric properties.

### 3.7. Thermogravimetric Analysis (TGA) 

**Figure 8:**
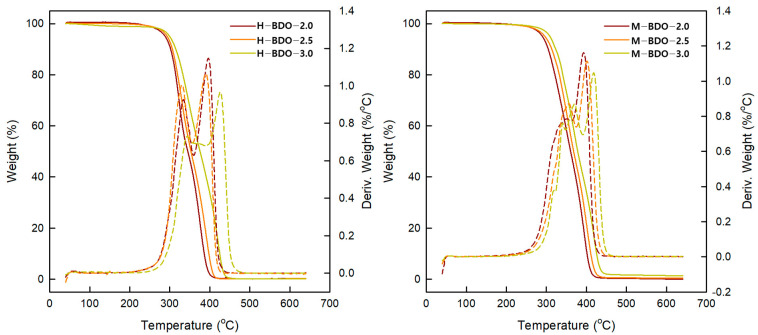
Thermogravimetric analysis curves and the respective derivative curves of bio-based TPUs.

The TGA curves for TPUs prepared under different conditions are shown in Figure 8, and the corresponding data are summarized in Table 9. The temperature of 5% weight loss (T5) is typically considered the onset decomposition temperature [41]. The results showed differences at the beginning of thermal degradation (T5) depending on the [NCO]/[OH] molar ratio. Furthermore, the molecular weight and T5 increased with the [NCO]/[OH] molar ratio. The T5 of the M-BDO-series was 2–9 °C higher than that of the H-BDO-series. In particular, the M-BDO-3.0 sample was characterized by the best thermal stability in the initial stage of decomposition at 300–310 °C. Thus, in M-BDO-3.0, a higher temperature is required to obtain 5 and 10% weight loss compared to that in other materials. Therefore, it is advantageous to use MDI as isocyanate from the viewpoint of thermal properties. Despite the presence of a single-step drop, there are two peaks in the derivative curve (%/°C; DTG) owing to the change in the curve slope as it descends. The first step at 330–370 °C is related to HS decomposition (T_HS_), and the second step around 390–426 °C is associated with SS decomposition (T_SS_) as shown in Figure 8 [67]. All TPUs prepared in this study showed this characteristic, and the weight loss rate was dependent on the [NCO]/[OH] molar ratio. With an increase in the [NCO]/[OH] molar ratio, the maximum weight loss rate for the first and second decomposition steps decreased. All TPUs with a higher HS content also exhibited a higher weight loss rate for the first decomposition step and a lower weight loss rate for the second decomposition step. In conclusion, an increase in the [NCO]/[OH] molar ratio leads to a corresponding increase in thermal stability. This relationship is reversed in T_HS_ at a higher temperature, suggesting a more cross-linked structure of the polymer. In addition, the materials obtained from aromatic isocyanate showed higher thermal stability than those obtained from aliphatic isocyanate. Thus, the type of isocyanate was revealed to have more effect on thermal stability. According to the literature, the initial stage of decomposition temperature of bio-based TPUs was revealed at 310 °C with fatty acid dimer-based polyester polyols [19] and 315 °C [30] with bio-based polyether polyols-poly(trimethylene glycol). Additionally, TPU from Bio-based polyester polyols synthesized using esterification with azelaic acid, sebacic acid, and 1,3-propanediol showed 260 °C of the initial stage of decomposition temperature [68]. In the results of DTG peaks of bio-based TPU, Carmen et al. [69] using dimer acid-based polyol described the temperature range of around 311–360 °C and 390–440 °C, Paulina et al. [70] with the poly(propylene succinate)s showed the temperature of 5% mass loss at ca. 320 °C, and T_HS_/T_SS_ at 384.7/427.3 °C. These results represented that bio-based TPU in this study had similar thermal stability compared to the other bio-TPU.

### 3.8. Mechanical Properties

**Figure 9:**
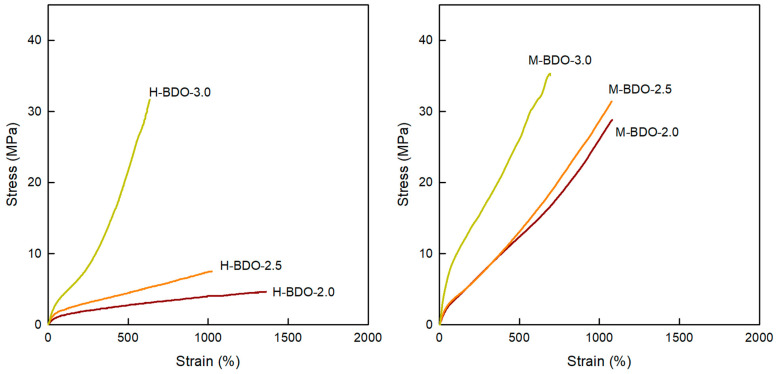
Tensile stress-strain curves of the bio-based TPUs.

Figure 9 and Table 10 illustrate the mechanical properties (initial modulus, tensile strength, and elongation at break) of all the samples at room temperature and hardness (Shore A). The mechanical performances of the TPUs are closely related to their compositions; the HS can act as physical cross-links and reinforcing units, while the SS is responsible for the material flexibility owing to the long linear polyol chain. From the data in Table 7, it can be concluded that the bio-based thermoplastic poly(ether-urethane)s prepared with the highest [NCO]/[OH] molar ratio has the highest HS content. The initial modulus values of the H-BDO- and M-BDO-series ranged from 12.20–42.40 MPa and 35.25–67.07 MPa, respectively. Therefore, the initial modulus values increased with the amount of HS content in the polymer, which was associated with higher stiffness and content of HS. Similar relationships were demonstrated for all prepared samples with respect to tensile strength and elongation at break. The increase in the HS content from 36.9 to 45.5 wt% resulted in a corresponding increase in the tensile strength from 4.61 to 31.61 MPa. The increase in the [NCO]/[OH] molar ratio also led to higher tensile strength. The HS content determines the tensile strength, while the SS content relates to the elongation at break values. The elongation at break is also related to the decrease in the chain mobility, and the permanent set after the break suggests the possibility of macromolecular chains returning to the states in which they were before the test. M-BDO-2.0 showed high elongation at break (1081%) and tensile strength values above 29 MPa. Moreover, M-BDO-3.0 showed higher hardness (Shore A), and the hardness value increased as the content of the HSs increased; hardness also increased with increasing [NCO]/[OH] molar ratio. The samples of M-BDO-2.0, M-BDO-2.5, and M-BDO-3.0 showed elongation at break values of 1316.8, 629.9, and 635.7%, respectively. These differences in the elongation at break are related to the decreases in the chain mobility, which was confirmed for the H-BDO-series by shifting the T_g_ to higher values. Hardness is closely connected with the cross-link density in HS, which decreased the elasticity of the samples and led to rigid materials. The MDI-based samples had the highest hardness, with values ranging from 79 to 86 Shore A. The stiffness of the materials increased as the cross-link density increased.

### 3.9. Micro-Phase Separation Characteristics

**Figure 10:**
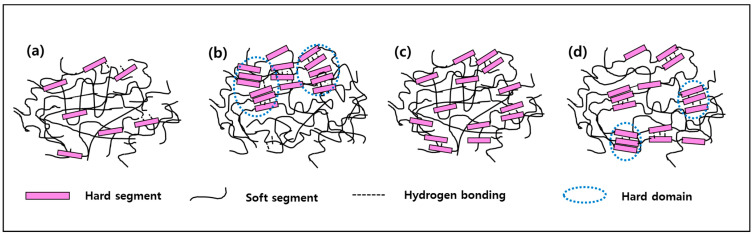
Schematic diagram of micro-phase separation in TPUs containing different conditions: (**a**) a disassociated structure of phase-mixed between HS and SS, (**b**) a hydrogen-bonded structure phase-separated between HS and SS that formed one-sided hard domains, (**c**) hydrogen-bonded structure of phase-mixed between the HS and SS, and (**d**) hydrogen-bonded structure of phase-separated between the HS and SS formed hard domains.

Based on the results discussed above (FT-IR, AFM, DSC, etc.), four types of micro-phase separation in a two-phase system are suggested for the HS dispersed in the SS matrix, as shown in Figure 10. The figure shows (a) a disassociated structure of phase-mixed between HS and SS, (b) a hydrogen-bonded structure phase-separated between HS and SS that formed one-sided hard domains, (c) hydrogen-bonded structure of phase-mixed between the HS and SS, and (d) hydrogen-bonded structure of phase-separated between the HS and SS that formed dispersed hard domains. Due to this, H-BDO-2.0 is expected to exhibit the (a)-form in Figure 10 among the phase-separated forms. The (a)-form can be regarded as a phase-separated form when phase separation is not accomplished completely. The AFM image showed an exceptionally smooth surface with low roughness, lower DPS values, and tensile strength (4.61 MPa). In the case of H-BDO-2.5 and H-BDO-3.0, because no significant difference was observed in the DPS values, a phase-mixed system between HS and SS is expected to show the (c)-form. In particular, visible softening of the materials based on the tensile test was observed in the case of the H-BDO-series, which also suggested a less ordered phase among the structures. This result is different compared to other thermoplastic elastomers that often show a decrease of the rubbery plateau with increasing temperature due to incomplete phase separation or partial melting of the HS. In DSC, the slight increase in T_g_ observed for the MDI-based TPU suggests the presence of a relatively large amount of HS mixed within the soft domain, resulting in a higher degree of microphase separation for this TPU series caused by the increase in the molecular weight and hydrogen-bonded hard domain. These results are in agreement with the FT-IR spectra in the C=O region, where free and hydrogen-bonded carbonyl groups are involved. The formation of the hard domain and the T_m_ of the hard phase is drastically affected by the HS length distribution. Researchers [19,46] have reported that the properties of TPUs can be improved using uniform HS and have a more complete-phase separation. The disordering in the HS and the partial miscibility of the hard phase in the SS are also confirmed by the decrease in the hard domain. [47,48]. The difference in the behavior of T_m_ of the TPU materials suggests that the TPUs have different physical origins. The lower temperatures can be attributed to the melting of the less-ordered structures or suitable SSs, whereas the higher temperatures are associated with greater-order structures. The structural proposal in Figure 8 was supported by the segment structure of TPU, which was used as a guideline to elucidate the morphology of these polymers further. Based on this, the M-BDO-2.0 sample shows the (b)-form with low T_m_ of HS despite the high DPS value. The sample of M-BDO-2.5 and M-BDO-3.0 may demonstrate the (d)-form with the AFM images, which can be confirmed with high DPS values, T_m_ of HS (231.6 °C), rubbery plateau (73.9–170 °C), and tensile strength (35.0 MPa) from the FT-IR, DSC, and DMA results.

## 4. Conclusions

Recently, there has been increased interest in developing bio-based polyols and TPUs owing to environmental issues, which will lead to a growing demand for materials with soft and strong properties. In order to meet these demands of the times, this study aimed to synthesize a bio-based TPU. We used bio-based PO3G as a polyol, MDI, and H_12_MDI as the isocyanate and bio-based BDO as the chain extender. Bio-based TPUs with [OH]/[NCO]/[OH] molar ratios ranging from 1:2:1 to 1:3:1 was successfully synthesized using a solvent-free one-shot process. This study investigated the effect of the equivalence ratio and isocyanate type on the compositions and properties of the HS and SS of the synthesized TPUs and then suggested four types of micro-phase separation of the HS and SS. The M_n_ and M_w_ of TPUs were in the ranges 37,723–112,117 and 70,953–236,689, respectively. The M_w_ of TPUs strongly increased as the [NCO]/[OH] molar ratio increased. The DPS also increased, and this result is expected to affect the thermal and physical properties and the micro-phase separation characteristics of the prepared film. When the HS content was increased by varying the [NCO]/[OH] molar ratio, the HS domains became larger; the roughness and the extent of phase separation increased, and the hard domain structures became more pronounced and visible in the sample morphology in the 3D AFM images. Considering the isocyanate type, the M-BDO-series showed higher average molecular weight and DPS than those of the H-BDO-series, despite having a lower content of HS. Consequently, the M-BDO-series had better-ordered, stronger, and more stable HS domains. The M-BDO-series with a high hard domain content showed a more rounded hilly break surface containing large globules and a high roughness value in the 3D AFM images. The SS T_g_, SS T_m_, HS T_g_, and HS T_m_ of M-BDO_3.0 were observed at −44.3, 80.4, 157.3, and 231.57 ℃, respectively, in the DSC analysis. The rubbery plateau was extended and exhibited a higher rubbery modulus and sharper peak, which was determined by DMA. According to TGA, the materials obtained from aromatic isocyanate showed higher thermal stability than those obtained from aliphatic isocyanate. In addition, the mechanical properties of the M-BDO-series were higher compared to those of the H-BDO-series. With respect to the micro-phase separation forms, H-BDO-2.0 is expected to show the (a)-form, indicating that phase separation is not accomplished completely because of the exceptionally smooth surface observed in AFM, with low roughness, lower DPS values, and tensile strength. H-BDO-2.5 and H-BDO-3.0 represented the (c)-form based on the tensile test and AFM, which also suggested a less-ordered phase among the micro-phase structures. The M-BDO-2.0 sample represented the (b)-form with low T_m_ of HS despite the high DPS value, while M-BDO-2.5 and M-BDO-3.0 can relate to the (d)-form with the AFM images, which can be confirmed with the high DPS values, T_m_ of HS, rubbery plateau, and tensile strength. The previous results showed that when an aromatic isocyanate (MDI) was used in the synthesis of TPU with a bio-based ether polyol, a soft sample with excellent thermal and physical properties could be obtained. In the future, we would like to investigate whether these TPU samples can be used as a shape memory polymer for 4D printing filaments.

## Figures and Tables

**Table 1:** The formulation design of bio-based TPU samples.

Sample	Diisocyanate	OH/NCO/OH	Hard Segment (HS) Content (wt%) ^a^	Content of Bio-Based Sources (wt%) ^b^
H-BDO-2.0	H_12_MDI	1:2.0:1	36.9	63
H-BDO-2.5	H_12_MDI	1:2.5:1	41.5	59
H-BDO-3.0	H_12_MDI	1:3.0:1	45.5	55
M-BDO-2.0	MDI	1:2.0:1	33.3	67
M-BDO-2.5	MDI	1:2.5:1	37.6	62
M-BDO-3.0	MDI	1:3.0:1	41.4	59

^a^ Hard segment concentration is defined as the ratio of the mass of non-polyol components to the total mass; ^b^ Bio-content is defined as the ratio of the mass of bio-based components to the total mass.

**Table 2:** Characteristics, structures, and molecular weights of the pure materials used in bio-based TPU synthesis.

Reagent Name	Supplier	Description	Molecular Structure
Polytrimethylene ether glycol (PO3G)	SK chemical, Korea	100% bio-based polyether polyol (1,3-propanediol based) by corn oil M_w_ 1000 g/mol, hydroxyl number = 53.4–59.0, T_m_ = 16–18 °C	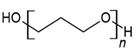
Dicyclohexylmethane diisocyanate (H_12_MDI)	Sigma Aldrich, Germany	Aliphatic diisocyanate, M_w_ 262.35 g/mol	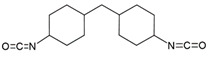
4,4′-Diphenylmethane diisocyanate (MDI)	Sigma Aldrich, Germany	Aromatic diisocyanate, M_w_ 250.25 g/mol	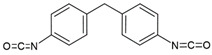
1,4-Butanediol (BDO)	Sigma Aldrich, Germany	Chain extender, M_w_ 90.122 g/mol	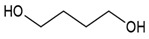
Dibutylin dilaurate (DBTDL)	Sigma Aldrich, Germany	Catalyst, M_w_ 631.56 g/mol	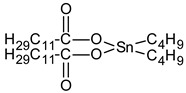

**Table 3:** Average molecular weight (M_n_ and M_w_) and polydispersity index (PDI) of bio-based TPUs.

Sample	M_n_	M_w_	PDI
H-BDO-2.0	37,723	70,953	1.88
H-BDO-2.5	40,375	72,013	1.78
H-BDO-3.0	112,117	236,689	2.11
M-BDO-2.0	57,476	112,220	1.95
M-BDO-2.5	60,829	153,285	2.51
M-BDO-3.0	n.m. ^a^	n.m.	n.m.

^a^: n.m. = not measured.

**Table 4:** Deconvolution of the FT-IR absorbance bands in the 1750–1680 cm^−1^ range that occurred in the prepared TPUs.

Sample	Hard Segment (HS)	Absorption Intensity of C=O Band		R	Degree of Phase Separation (DPS)	Degree of Phase Mixing (DPM)
		Free C=O 1730 cm^−1^	H-Bonded C=O 1700 cm^−1^			
H-BDO-2.0	36.9	29.37	35.88	1.22	0.550	0.450
H-BDO-2.5	41.5	27.87	35.53	1.24	0.553	0.447
H-BDO-3.0	45.5	29.80	37.16	1.25	0.555	0.445
M-BDO-2.0	33.3	30.12	40.53	1.35	0.574	0.426
M-BDO-2.5	37.6	30.55	41.89	1.37	0.578	0.422
M-BDO-3.0	41.4	30.08	42.00	1.40	0.583	0.417

**Table 5:** Characterization of phase images of TPU films.

Sample	Surface Area (μm^2^)	R_q_ (nm) ^a^	R_a_ (nm) ^b^	R_max_ (nm) ^c^
H-BDO-2.0	401	29	23	277
H-BDO-2.5	437	257	177	1604
H-BDO-3.0	441	294	228	2177
M-BDO-2.0	414	227	178	1872
M-BDO-2.5	415	305	248	2328
M-BDO-3.0	408	417	347	4243

Surface area: total area of the examined sample surface. Mean: average of all the Z values within the enclosed area. ^a^ R_q_ (rms): standard deviation of the Z values within the given area. ^b^ R_a_ (mean roughness): mean value of the surface relative to the center place. ^c^ R_max_ (maximum height): difference in height between the highest and the lowest points on the surface relative to the mean plane.

**Table 6:** Peak analysis data for X-ray diffraction peaks.

Sample	Peak 1 (θ = 19.40)		Peak 2 (θ = 23.46)	
	Intensity	FWHM ^a^	Intensity	FWHM
H-BDO-2.0	4858.01	6.00	752.15	4.44
H-BDO-2.5	4664.34	5.85	713.71	4.98
H-BDO-3.0	5046.76	5.96	609.42	6.53
M-BDO-2.0	3354.74	6.55	914.39	4.07
M-BDO-2.5	5069.32	6.23	820.04	6.16
M-BDO-3.0	5291.63	5.97	591.92	6.36

^a^ full width at half maximum (FWHM): peak width.

**Table 7:** A summary of DSC results of the bio-based TPUs.

Sample	SS T_g_ (°C)	SS T_m_ (°C)	HS T_g_ (°C)	HS T_m_ (°C)
H-BDO-2.0	−55.4	71.6	-	195.8
H-BDO-2.5	−55.0	80.3	145.6	217.4
H-BDO-3.0	−52.4	83.5	166.0	219.6
M-BDO-2.0	−47.7	67.1	136.3	160.1
M-BDO-2.5	−46.0	69.3	151.8	183.8
M-BDO-3.0	−44.3	80.4	157.3	231.6

**Table 8:** A summary of dynamic mechanical analysis results of the bio-based TPUs.

Sample	T_g_ (°C)	G′_25_(MPa)	T_flex_ (°C)	T_flow_ (°C)
H-BDO-2.0	−54.8	0.65	-	-
H-BDO-2.5	−49.3	13.18	-	77.7
H-BDO-3.0	−40.6	46.34	80.1	138.0
M-BDO-2.0	−37.2	1.20	60.2	92.5
M-BDO-2.5	−37.2	5.05	94.2	102.0
M-BDO-3.0	−36.8	54.39	73.9	170.0

**Table 9:** Thermal decomposition characteristics of the bio-based TPUs.

Sample	T5% (°C)	T10% (°C)	T50% (°C)	T_HS_ (°C)	T_SS_ (°C)
H-BDO-2.0	288.8	302.4	345.3	334.7	396.4
H-BDO-2.5	294.0	306.7	353.2	332.4	390.5
H-BDO-3.0	300.0	315.3	375.8	344.2	426.0
M-BDO-2.0	290.0	304.7	360.7	351.3	391.6
M-BDO-2.5	296.5	313.5	368.0	356.0	402.2
M-BDO-3.0	308.9	323.4	378.2	371.5	418.9

**Table 10:** Mechanical properties and hardness of the bio-based TPUs.

Sample	Initial Modulus (MPa)	Tensile Strength(MPa)	Elongation (%)	Energy (J)	Hardness (Shore A)
H-BDO-2.0	12.20	4.61	1316.80	0.13	70
H-BDO-2.5	22.23	7.48	1024.50	0.14	79
H-BDO-3.0	42.40	31.61	635.70	1.01	85
M-BDO-2.0	35.25	28.84	1080.90	2.09	76
M-BDO-2.5	37.27	31.42	1076.90	3.55	77
M-BDO-3.0	67.07	35.04	693.90	3.24	86

## Data Availability

The data presented in this study are available on request from the corresponding author.

## References

[1] Oh J., Kim Y.K., Hwang S.H., Kim H.C., Jung J.H., Jeon C.H., Kim J., Lim S.K. (2022). Synthesis of Thermoplastic Polyurethanes Containing Bio-Based Polyester Polyol and Their Fiber Property. Polymers.

[2] Duval A., Sarbu A., Dalmas F., Albertini D., Averous L. (2022). 2, 3-Butanediol as a Biobased Chain Extender for Thermoplastic Polyurethanes: Influence of Stereochemistry on Macromolecular Architectures and Properties. Macromolecules.

[3] Gao Z., Wang Z., Liu Z., Fu L., Li X., Eling B., Pöselt E., Schander E., Wang Z. (2022). Hard block length distribution of thermoplastic polyurethane determined by polymerization-induced phase separation. Polymer.

[4] Zhu Y., Romain C., Williams C.K. (2016). Sustainable polymers from renewable resources. Nature.

[5] Yilgör I., Yilgor E., Das S., Wilkes G.L. (2009). Time-dependent morphology development in segmented polyetherurea copolymers based on aromatic diisocyanates. J. Polym. Sci. Part B Polym. Phys..

[6] Kasprzyk P., Głowińska E., Parcheta-Szwindowska P., Rohde K., Datta J. (2021). Green TPUs from Prepolymer Mixtures Designed by Controlling the Chemical Structure of Flexible Segments. Int. J. Mol. Sci..

[7] Zhang C., Garrison T.F., Madbouly S.A., Kessler M.R. (2017). Recent advances in vegetable oil-based polymers and their composites. Prog. Polym. Sci..

[8] Donati I., Travan A., Pelillo C., Scarpa T. (2009). Polyol Synthesis of Silver Nanoparticles: Mechanism of Reduction by Alditol Bearing Polysaccharides. Biomacromolecules.

[9] Hakima A., Nassar M., Emam A., Sultan M. (2011). Preparation and characterization of rigid polyurethane foam prepared from sugar-cane bagasse polyol. Mater. Chem. Phys..

[10] Lu M.Y., Surányi A., Viskolcz B., Fiser B. (1991). Molecular design of sugar-based polyurethanes. Croat. Chem. Acta.

[11] Li Y., Luo X., Hu S. (2015). Lignocellulosic Biomass-Based Polyols for Polyurethane Applications Bio-Based Polyols and Polyurethanes.

[12] Zlatanić A., Lava C., Zhang W., Petrović Z.S. (2004). Effect of structure on properties of polyols and polyurethanes based on different vegetable oils. J. Polym. Sci. Part B Polym. Phys..

[13] Sawpan M. (2018). Polyurethanes from vegetable oils and applications: A review. J. Polym. Res..

[14] Petrovic Z., Yang L., Zlatanic A., Zhang W., Javni I. (2007). Network structure and properties of polyurethanes from soybean oil. J. Appl. Sci..

[15] Gurunathan T., Mohanty S., Nayak S.K. (2015). Isocyanate terminated castor oil-based polyurethane prepolymer: Synthesis and characterization. Prog. Org. Coat..

[16] Alagi P., Hong S.C. (2015). Vegetable oil-based polyols for sustainable polyurethanes. Macromol. Res..

[17] Chuayjuljit S., Maungchareon A., Saravari O. (2010). Preparation and properties of palm oil-based rigid polyurethane nanocomposite foams. J. Reinf. Plast. Compos..

[18] Shen Y., He J., Xie Z., Zhou X., Fang C., Zhang C. (2019). Synthesis and characterization of vegetable oil based polyurethanes with tunable thermomechanical performance. Ind. Crops Prod..

[19] Ruan M., Luan H., Wang G., Shen M. (2019). Bio-polyols synthesized from bio-based 1,3-propanediol and applications on polyurethane reactive hot melt adhesives. Ind. Crops Prod..

[20] Fakirov S. (2005). Handbook of Condensation Thermoplastic Elastomer.

[21] Holden G., Kricheldorf R., Quirk P. (2004). Thermoplastic Elastomer.

[22] Harrell L.J. (1969). Segmented polyurethanes. Properties as function of segment size and distribution. Macromolecules.

[23] Delebecq E., Pascault J.P., Boutevin B., Ganachaud F. (2013). On the versatility of urethane/urea bonds: Reversibility, blocked isocyanate, and non-isocyanate polyurethane. Chem. Rev..

[24] Charlon M., Heinrich B., Matter Y., Couzigné E., Donnio B., Avérous L. (2014). Synthesis, structure, and properties of fully biobased thermoplastic polyurethanes, obtained from a diisocyanate based on modified dimer fatty acids, and different renewable diols. Eur. Polym. J..

[25] Gaymans R.J. (2011). Segmented copolymers with monodisperse crystallizable hard segments: Novel semi-crystalline materials. Prog. Polym. Sci..

[26] Wang Y., Ma R., Li H., Hu S., Gao Y., Liu L., Zhang L. (2022). Effect of the content and strength of hard segment on the viscoelasticity of the polyurethane elastomer: Insights from molecular dynamics simulation. Soft Matter.

[27] Kang S., Ji Z., Tseng L., Turner S., Villanueva D., Johnson R., Albano A., Langer R. (2018). Design and Synthesis of Waterborne Polyurethanes. Adv. Mater..

[28] Biemond G. (2006). Hydrogen Bonding in Segmented Block Copolymer. Ph.D. Thesis.

[29] Kasprzyk P., Benes H., Donato R.K., Datta J. (2020). The role of hydrogen bonding on tuning hard-soft segments in bio-based thermoplastic poly(ether-urethane)s. J. Clean. Prod..

[30] Niesten M., Feijen J., Gaymans R.J. (2000). Synthesis and properties of segmented copolymers having aramid units of uniform length. Polymer.

[31] Van der Schuur M., Gaymans R.J. (2006). Segmented block copolymers based on poly(propylene oxide) and monodisperse polyamide-6,T segments. J. Polym. Sci. Part A Polym. Chem..

[32] Odian G. (2004). Step Polymerization, in Principles of Polymerization.

[33] Kirpluks M., Cabulis U., Ivdre A., Kuranska M., Zieleniewska M., Auguscik M. (2016). Mechanical and thermal properties of high-density rigid polyurethane foams from renewable resources. J. Renew. Mater..

[34] Li X.X., Sohn M.H., Cho U.R. (2019). Synthesis and Properties of Bio-Thermoplastic Polyurethanes with Different Isocyanate Contents. Elastomers Compos..

[35] Coleman M., Lee K., Skrovanek D., Painter P. (1986). Hydrogen bonding in polymers. 4. Infrared temperature studies of a simple polyurethane. Macromolecules.

[36] Lei W., Fang C., Zhou X., Cheng Y., Yang R., Liu D. (2017). Morphology and thermal properties of polyurethane elastomer based on representative structural chain extenders. Thermochim. Acta.

[37] Gorna K., Polowinski S., Gogolewski S. (2002). Synthesis and characterization of biodegradable poly(e-caprolactone urethane)s. I. Effect of the polyol molecular weight, catalyst, and chain extender on the molecular and physical characteristics. J. Polym. Sci. Part A Polym. Chem..

[38] Xiaozhen Y., Decai Y., Hsu S.L., Meuse C.W. (1992). Spectroscopic analysis of ordering and phase-separation behavior of model polyurethanes in a restricted geometry. Macromolecules.

[39] Elwell M.J., Ryan A.J., Grünbauer H.C., Lieshout V. (1996). In-situ studies of structure development during the reactive processing of model flexible polyurethane foam systems using FT-IR spectroscopy, synchrotron SAXS, and rheology. Macromolecules.

[40] Głowińska E., Datta J. (2016). Bio polyetherurethane composites with high content of natural ingredients: Hydroxylated soybean oil based polyol, bio glycol and microcrystalline cellulose. Cellulose.

[41] Ryan A.J., Willkomm W.R., Bergstrom T.B., Macosko C.W., Koberstein J.T., Yu C.C., Russell T.P. (1991). Dynamics of (Micro)phase separation during fast, bulk copolymerization: Some synchrotron SAXS experiments. Macromolecules.

[42] Król P. (2007). Synthesis methods, chemical structures and phase structures of linear polyurethanes. Properties and applications of linear polyurethanes in polyurethane elastomers, copolymers and ionomers. Prog. Mater. Sci..

[43] Saralegi A., Rueda L., Fernández-D’Arlas B., Mondragon A., Eceiza C. (2013). Thermoplastic polyurethanes from renewable resources: Effect of soft segment chemical structure and molecular weight on morphology and final properties. Polym. Int..

[44] Suzuki T., Shibayama M., Hatano K., Ishii M. (2009). [NCO]/[OH] and acryl-polyol concentration dependence of the gelation process and the microstructure analysis of polyurethane resin by dynamic light scattering. Polymer.

[45] Kasprzyk P., Datta J. (2018). Effect of Molar Ratio [NCO]/[OH] Groups during Prepolymer Chains Extending Step on the Morphology and Selected Mechanical Properties of Final Bio-Based Thermoplastic Poly ( Ether-Urethane) Materials. Polym. Eng. Sci..

[46] Versteegen M., Sijbesma P., Meijer W. (2005). Synthesis and characterization of segmented copoly(ether urea)s with uniform hard segments. Macromolecules.

[47] Flory J. (1942). Thermodynamics of High Polymer Solutions. J. Chem. Phys..

[48] Verstraete G., Van Renterghem J., Van Bockstal P.J., Kasmi S., De Geest B.G., De Beer T., Vervaet C. (2016). Hydrophilic thermoplastic polyurethanes for the manufacturing of highly dosed oral sustained release matrices via hot melt extrusion and injection molding. Int. J. Pharm..

[49] Kim H.D., Huh J.H., Kim E.Y., Park C.C. (1998). Comparison of properties of thermoplastic polyurethane elastomers with two different soft segments. J. Appl. Polym. Sci..

[50] Tao Y., Hasan A., Deeb G., Hu C., Han H. (2016). Rheological and mechanical behavior of silk fibroin reinforced waterborne polyurethane. Polymers.

[51] Lluch C., Esteve-Zarzoso B., Bordons A., Lligadas G., Ronda J.C., Galia M., Cádiz V. (2014). Antimicrobial polyurethane thermosets based on undecylenic acid: Synthesis and evaluation. Macromol. Biosci..

[52] Li H., Mahmood N., Ma Z., Zhu M., Wang J., Zheng J., Yuan Z., Wei Q., Xu C. (2017). Preparation and characterization of bio-polyol and bio-based flexible polyurethane foams from fast pyrolysis of wheat straw. Ind. Crops Prod..

[53] Sheth J.P., Klinedinst D.B., Wilkes G.L., Yilgor I., Yilgor E. (2005). Role of chain symmetry and hydrogen bonding in segmented copolymers with monodisperse hard segments. Polymer.

[54] Kultys A., Rogulska M., Pikus S., Skrzypiec K. (2009). The synthesis and characterization of new thermoplastic poly(carbonate-urethane) elastomers derived from HDI and aliphatic-aromatic chain extenders. Eur. Polym. J..

[55] Kojio K., Kugumiya S., Uchibaq Y., Nishino Y., Furukawa M. (2009). The micro-separated structure of polyurethane bulk and thin films. Polym. J..

[56] Špírková M., Strachota A., Urbanová M., Baldrian J., Brus J., Šlouf M., Kuta A., Hrdlička Z. (2009). Structural and surface properties of novel polyurethane films. Mater. Manuf. Process..

[57] Wang C., Ma C., Mu C., Lin W. (2017). Tailor-made zwitterionic polyurethane coatings: Microstructure, mechanical property and their antimicrobial performance. RSC Adv..

[58] Špírková M., Pavličević J., Strachota A., Poreba R., Bera O., Kaprálková L., Baldrian J., Šlouf M., Lazić N., Budinski-Simendić J. (2011). Novel polycarbonate-based polyurethane elastomers: Composition–property relationship. Eur. Polym. J..

[59] Fuensanta M., Jofre-Reche J.A., Rodríguez-Llansola F., Costa V., Iglesias J.I., Martín-Martínez J.M. (2017). Structural characterization of polyurethane ureas and waterborne polyurethane urea dispersions made with mixtures of polyester polyol and polycarbonate diol. Prog. Org. Coat..

[60] Jeffrey T.K., Adam F. (1992). Galambos Multiple melting in segmented polyurethane block copolymers. Macromolecules.

[61] Fernández-d’Arlas B., Jens B., Peter R., Pöselt E., Raphael, Berend T., Müller A. (2016). Tailoring the Morphology and Melting Points of Segmented Thermoplastic Polyurethanes by Self-Nucleation. Macromolecules.

[62] Saiani A., Daunch W.A., Verbeke H., Leenslag J.-W., Higgins J.S. (2001). Origin of Multiple Melting Endotherms in a High Hard Block Content Polyurethane. 1. Thermodynamic Investigation. Macromolecules.

[63] Saiani A., Rochas C., Eeckhaut G., Daunch W.A., Leenslag J.-W., Higgins J.S. (2004). Origin of Multiple Melting Endotherms in a High Hard Block Content Polyurethane. 2. Structural Investigation. Macromolecules.

[64] Saiani A., Novak A., Rodier L., Eeckhaut G., Leenslag J.-W. (2007). Higgins, J.S. Origin of Multiple Melting Endotherms in a High Hard Block Content Polyurethane:  Effect of Annealing Temperature. Macromolecules.

[65] Zhang L., Huang M.R., Yu J., Huang X., Dong R., Zhu J. (2014). Bio-based shape memory polyurethanes (Bio-SMPUs) with short side chains in the soft segment. J. Mater. Chem. A.

[66] Parcheta P., Datta J. (2018). Structure-rheology relationship of fully bio-based linear polyester polyols for polyurethane-Synthesis and investigation. Polym. Test..

[67] Chattopadhyay D.K., Webster D.C. (2009). Thermal stability and flame retardancy of polyurethanes. Prog. Polym. Sci..

[68] Sohn M.H., Li X.X., Cho U.R. (2019). Synthesis of Biomass-derived Polyurethane by Chain Extender Type. Elastomers Compos..

[69] Bueno-Ferrer C., Hablot E., del Carmen Garrigós M., Bocchini S., Averous L., Jiménez A. (2012). Relationship between morphology, properties and degradation parameters of novative biobased thermoplastic polyurethanes obtained from dimer fatty acids. Polym. Degrad. Stab..

[70] Paulina P., Ewa G., Janusz D. (2020). Effect of bio-based components on the chemical structure, thermal stability and mechanical properties of green thermoplastic polyurethane elastomers. Eur. Polym. J..

